# Micro-hydrogel Particles Consisting of Hyperbranched Polyamidoamine for the Removal of Heavy Metal Ions from Water

**DOI:** 10.1038/s41598-017-10066-x

**Published:** 2017-08-30

**Authors:** Sanghwa Lee, Youngsik Eom, Jeyoung Park, Jinhee Lee, Sang Youl Kim

**Affiliations:** 1 Department of Chemistry, Korea Advanced Institute of Science and Technology (KAIST), Daejeon, 34141 Republic of Korea; 2 Research Center for Bio-based Chemistry, Korea Research Institute of Chemical Technology (KRICT), Ulsan, 44429 Republic of Korea; 3 Advanced Materials and Chemical Engineering, University of Science & Technology (UST), Daejeon, 34113 Republic of Korea

## Abstract

A series of micro-hydrogel particles consisting of hyperbranched polyamidoamine (HPAMAM) without any supporting core materials was synthesized via the inverse suspension condensation polymerization of A_2_ and B_4_ monomers, *N,N*′-methylenebisacrylamide (**MBA**) and ethylenediamine (**EDA**). The particles were found to be highly effective when used to remove heavy metal ions, such as cadmium, copper, lead, nickel, zinc, and cobalt, from water, and they could be separated from the water by a simple filtration process. The results of this study demonstrate that crosslinked HPAMAM particles, which can be prepared by a simple and environmentally friendly process, are an attractive absorbent for water purification.

## Introduction

Water pollution by toxic heavy metal ions is a serious global nuisance owing to the harmful effects of heavy metals on the environment and human health^1, 2^. In order to address this issue, various methods for the removal of heavy metal ions in aqueous solutions, including precipitation, ion exchange, adsorption, membrane filtration, coagulation, and electrochemical treatments, have been developed^3–16^. Among these methods, adsorption techniques using adsorbent materials capable of capturing pollutants are easy to apply at a reasonable cost^3–11^. Specifically, chelating resins are an important category of adsorbent materials^3–9^. The majority of chelating resins consist of polystyrene beads, but a toxic chloromethylation reaction is necessary to make chelating resins from polystyrene^17, 18^. Accordingly, research related to the production of eco-friendly chelating resins has received much attention^19, 20^.

Polyamidoamine (PAMAM) dendrimers and hyperbranched polymers have attracted considerable interest in recent years. The highly branched structures together with a large number of functional groups in the main chains and end groups make these materials ideal for applications in host-guest encapsulation, nanoreactors, and delivery devices^21–30^. Amine and amide functional groups of PAMAM dendrimers can serve as nanoscale high-capacity containers for heavy metal ions such as Cu^2+^, Cd^2+^, and others^24–30^. However, PAMAM dendrimers require tedious multi-step synthesis to obtain a high molecular weight. Therefore, the analogous, hyperbranched polyamidoamine (HPAMAM), has been utilized in various categories in place of PAMAM dendrimers^31–33^. Drawbacks of PAMAM dendrimers and HPAMAM include their small size, the hydrodynamic diameter of the dendrimers, and the radius of gyration of the hyperbranched polymers, which is typically less than 10 nanometers^21, 25, 28^. Thus, additional processes such as ultrafiltration are necessary for separation from an aqueous solution^24–28^.

Generally, hyperbranched polymers can be synthesized via the one-step polymerization of an AB_x_ (X ≥ 2) monomer^34–36^. However, given that AB_x_ monomers includes different (A and B) functional groups with two types of reactivity, multiple synthesis routes must be carried out to form a monomer in the AB_x_ form. To overcome this problem, a method involving the polycondensation of a mixture of multifunctional monomers with a single type of reactive group with the slow feeding of one monomer into the other has been developed^36–38^. Although synthetic methods using a mixture of multifunctional monomers opens a way of preparing hyperbranched polymers from readily available multifunctional compounds, the polymerization of multifunctional monomer mixtures such as A_2_ and B_4_ monomers (A_2_ + B_4_ polycondensation) produces crosslinked polymers, even with the slow feeding of one monomer into the other, when the conversion reaches a gelation point (the critical extent of reaction).

In this study, we used both the advantages and limitations of the A_2_ + B_4_ polycondensation method for hyperbranched polymers to make micro-sized HPAMAM hydrogel particles through the dispersal of an aqueous HPAMAM solution obtained by A_2_ + B_4_ polycondensation into an immiscible liquid and then by carrying out the polycondensation until reaching the point of critical gelation^39–45^. This simple method allowed us to make micro-sized hydrogel particles which wholly consisted of hyperbranched polyamidoamine (HPAMAM) without any additional crosslinking reagents. The crosslinked HPAMAM hydrogel particles were found to be highly effective when used to remove heavy metal ions from an aqueous solution and could easily be separated from water by a simple filtration process. We also demonstrated that the properties of HPAMAM particles, in this case the particle size, gel fraction, swelling ratio, and absorption capacity for metal ions, could be tuned by controlling the monomer feed composition, the amount of stabilizer used, and the agitation speed.

## Results

**Figure 1:**
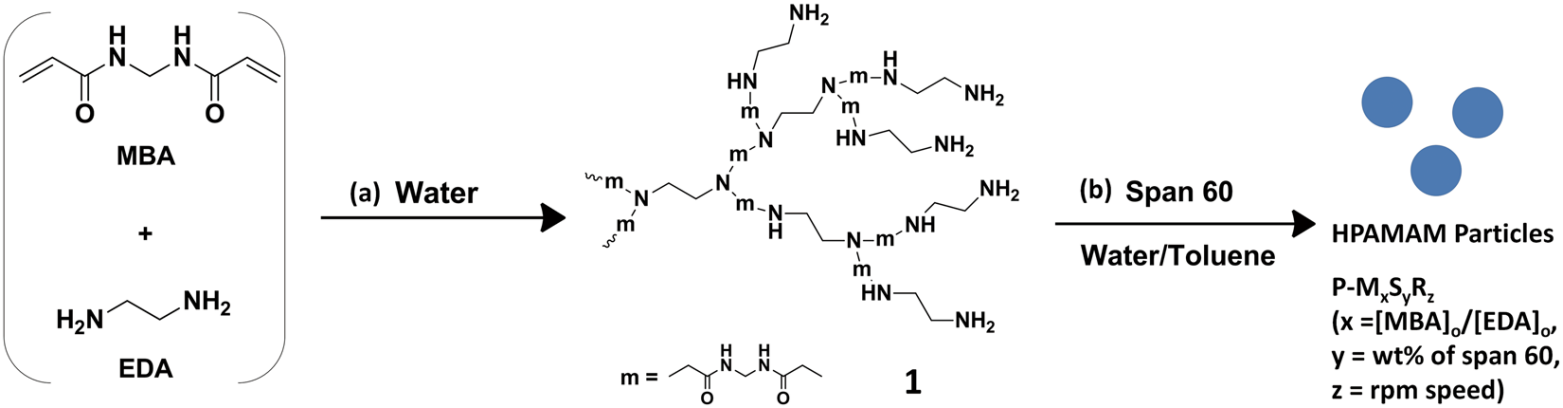
Synthesis of crosslinked hyperbranched polyamidoamine particles (**a**) in an aqueous solution, and (**b**) via inverse suspension polymerization.

**Figure 2:**
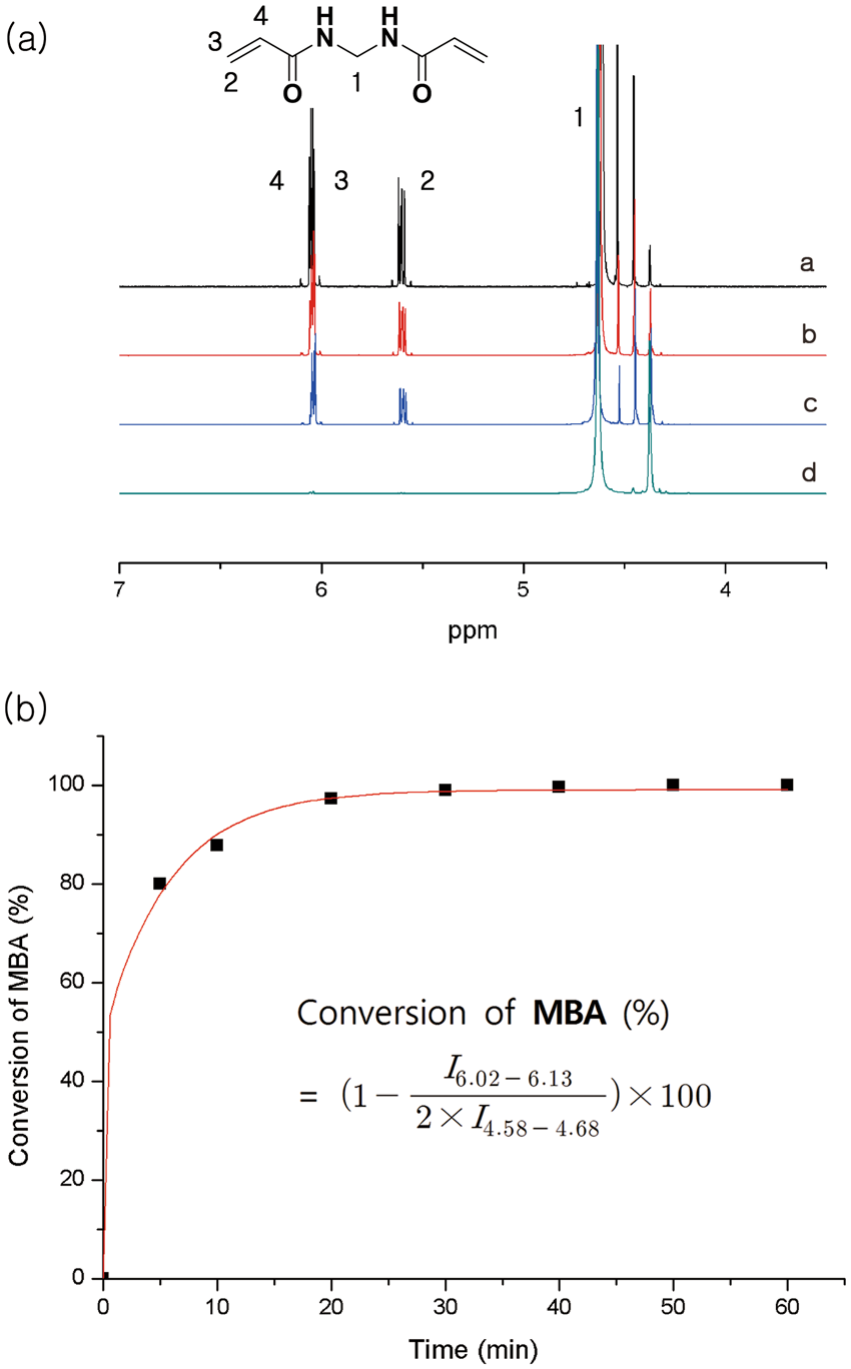
(**a**)^1^H NMR spectra of HPAMAM obtained at various polymerization times: (a) 0 min, (b) 5 min, (c) 10 min, and (d) 40 min (400 MHz, D_2_O), (**b**) polymerization time-dependent conversion of **MBA** monomer (inset: equation for the calculation of the conversion of **MBA**).

Michael addition reactions of commercially available A_2_ (*N,N*′-methylenebisacrylamide, **MBA**) and B_4_ (ethylenediamine, **EDA**) monomers were carried out in deionized (DI) water to investigate whether the synthesis of hyperbranched polyamidoamines is feasible, as shown in Fig. 1. Tentatively, a reaction of equal mol amounts of **MBA** and **EDA** (molar ratio of the reactive sites of **MBA** to **EDA** is 0.5) was attempted to consume all of the vinyl groups while preserving the primary amine groups as an end group. A water-insoluble polymer gel (**1**) was produced after 30 min of the simple mixing of the two monomers. Generally, the polymerization of a multifunctional monomer such as A_2_ and B_4_ produces crosslinked polymers, and the AB_2_ type of monomer is necessary to make a hyperbranched polymer. However, if one monomer (A_2_) is added slowly to the reaction mixture, the remaining reactive groups become only B, which prevents the formation of a gel-inducing species containing several A and B groups. Slow feeding of the A_2_ monomer (with a deficient concentration of vinyl groups in an aqueous solution) achieved by the relatively low solubility of **MBA** in water allows a preferential reaction of the vinyl group with readily available primary amines of **EDA** to form covalent bonds of the secondary amine. The formation of crosslinking points (i.e., tertiary amines) is suppressed in the early stage of polymerization mainly due to the low concentration of secondary amines as compared to that of the primary amines. However, the mixture of multifunctional monomers (A_2_ + B_4_) becomes a gel after a critical gelation point. The time-dependent conversion of the **MBA** monomer was monitored by assessing the ^1^H NMR spectra in the middle of the polymerization using D_2_O as a solvent to follow the reaction (Fig. 2).

The intensity of the peak around 6 ppm (*I*_6.02–6.13_) corresponding to the integral values of the protons (A) attached to the vinyl groups (6.02–6.13 ppm) decreased gradually compared to the intensity of the peak around 4.6 ppm (*I*_4.58–4.68_) corresponding to the integral values of the protons (B) of methylene (-CONH*CH*_*2*_CONH-, 4.5–4.7 ppm). The conversion reached 80% after 5 min of polymerization, 88% after 10 min and >99% after 40 min. It appears that the addition reaction occurred very rapidly in the initial period of polymerization and that the retro-Michael addition was negligible.

The critical gelation points predicted by Flory’s and Carothers’ equation for equal amounts of bifunctional monomer **MBA** and tetrafunctional monomer **EDA** are 0.82 and 1.00, respectively. Therefore, the gel formed above 82% but below 100% conversion, and gel formation was observed after 10 min at RT.

Based on the NMR analysis results, the preparation of micro-hydrogel particles which consisted of hyperbranched polyamidoamines was attempted via inverse suspension polymerization. The polymerization mixture in an aqueous solution was suspended within the organic phase which contains a stabilizer but without any initiators or cross-linking agents. Further conversion of the polymerization mixture (a homogeneous precursor solution) is expected to induce a gel through crosslinking when it reaches a critical gelation point. Samples of HPAMAMs are designated as **P-M**_**x**_ **S**_**y**_ **R**_**z**_, where x, y, and z denote the molar feed ratio of **MBA** to **EDA**, the weight concentration of Span 60, and the rpm speed of agitation, respectively. To investigate the effects of experimental parameters on the morphology, absorption properties, and other characteristics, the crosslinked HPAMAM particles were prepared under various reaction conditions (Table S1, ESI).

The representative micro-hydrogel particle **P-M**_**8/8**_ **S**_**0.5**_ **R**_**1k**_ was characterized by FT-IR and thermogravimetric analysis (TGA). The FT-IR spectra showed peaks of –*NH*- stretching (3000~3350 cm^−1^), -*CO*- stretching (1649 cm^−1^), and -CO*NH*- stretching (1537 cm^−1^), respectively. The *C*=*CH*_*2*_ stretching peak (3080 cm^−1^) of **MBA** nearly disappeared (Fig. S1, ESI). The 5% weight loss temperature of **P-M**_**8/8**_ **S**_**0.5**_ **R**_**1k**_ was 240 °C (Fig. S2, ESI).

## Discussion

The cross-linking density of the particles is closely related to their properties as absorbent media for water treatment applications, as this parameter affects the swelling ratio, heavy metal absorption capabilities, and degree of solvent resistance. The cross-linking density of HPAMAM particles is easily controlled by changing the initial feed ratio of the two monomers. For practical applications, the stability of particles in an aqueous solution is also important to minimize the loss of the capacity to absorb metal ions. Dried HPAMAM particles were placed in distilled water and stirred for at least two days to dissolve any unreacted monomers and oligomers, after which the HPAMAM particles were filtered and dried under a vacuum. The optimum feed ratio of the monomers was determined by measuring the gel fraction (Table S1 and Fig. S3, ESI). The soluble fraction of particles decreased to saturated values (less than 3 wt% of the particles) when the molar feed ratio of **MBA** to **EDA** approached 1.5. It seems that the cross-linking density of the HPAMAM particles obtained at a molar feed ratio of 1.5 is sufficiently stable in water to be recycled. From this study, investigations of the effect of a stabilizer and the speed of agitation were carried out with HPAMAM particles prepared with a molar feed ratio of 1.5 (**MBA** to **EDA**).

**Figure 3:**
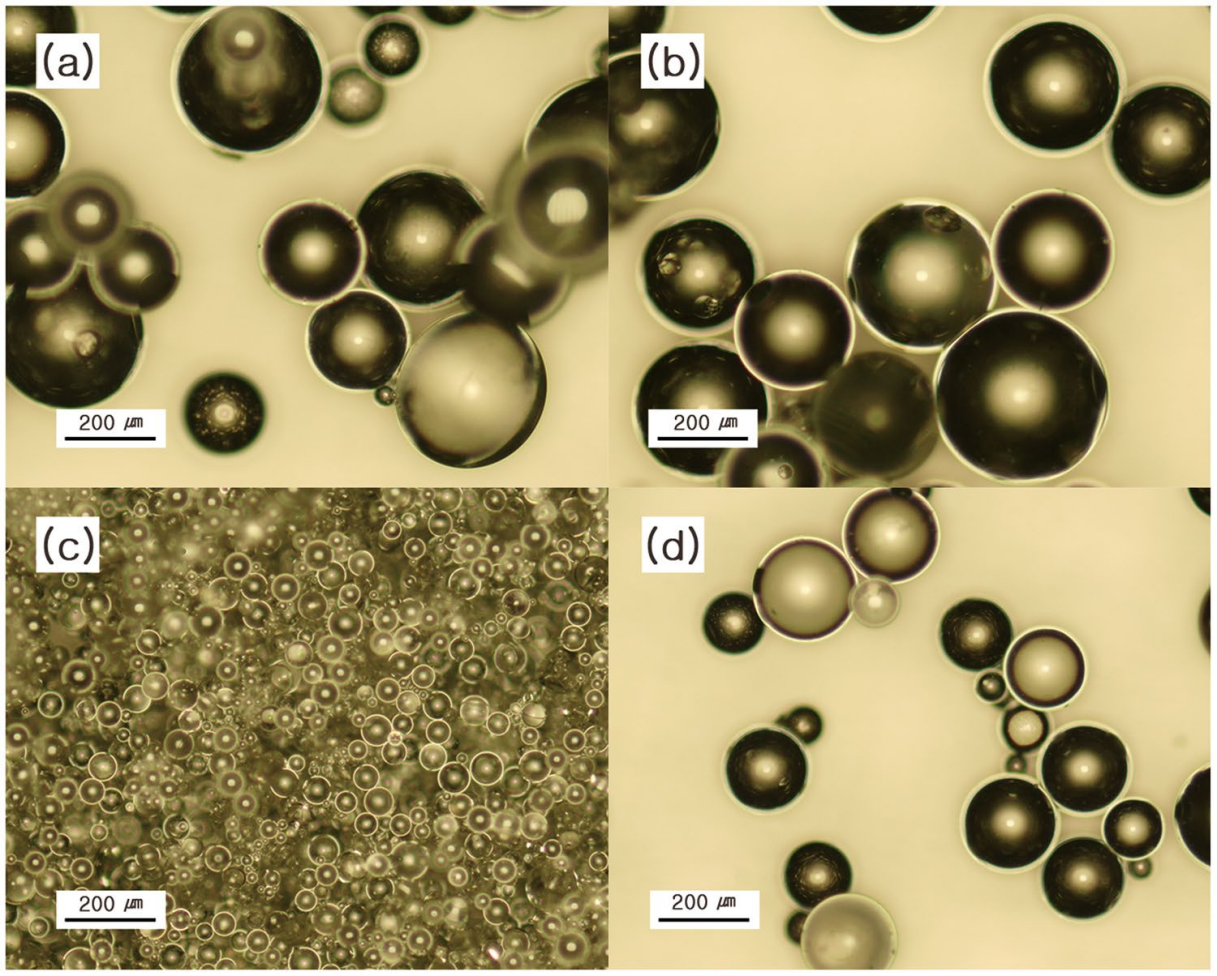
Optical images of (**a**) P-M_8/8_S_0.5_R_1k_, (**b**) P-M_12/8_S_0.5_R_1k_, (**c**) P-M_12/8_S_5.0_R_1k_, and (**d**) P-M_12/8_S_0.5_R_1.5k_.

The morphology of the synthesized micro-hydrogel particles was investigated with an optical microscope and a scanning electron microscope (SEM), as shown in Fig. 3 and Figs S4–7 (ESI). Interestingly, the diameter of spherical particles did not change regardless of the feed ratio of the monomers (50~250 μm), indicating that the particle size was not affected by the cross-linking density. However, increasing the weight concentration of the stabilizer or the speed of agitation decreased the diameter, as expected.

The swelling ratio of the particles (g/g) was obtained by measuring the weight gain of the particles after soaking them in distilled water. The Cu^2+^ absorption capacity (g/g) was measured by analyzing the change in the concentration of copper in the CuCl_2_ solution (10,000 ppm) with an inductively coupled plasma optical emission spectrometer (ICP-OES). As expected, the swelling ratio of the particles decreased with an increase in the amount of **MBA** because more cross-linked HPAMAM particles were formed as the stoichiometric ratio of the two functional groups (amine and vinyl groups) became two (one amine group can react with two acrylic groups; Fig. S8a, ESI). The Cu^2+^ absorption capacity also decreased with an increase in the amount of **MBA** up to the stoichiometric point, presumably due to the smaller number of free amine groups in the particles (Fig. S8b, ESI).

**Figure 4:**
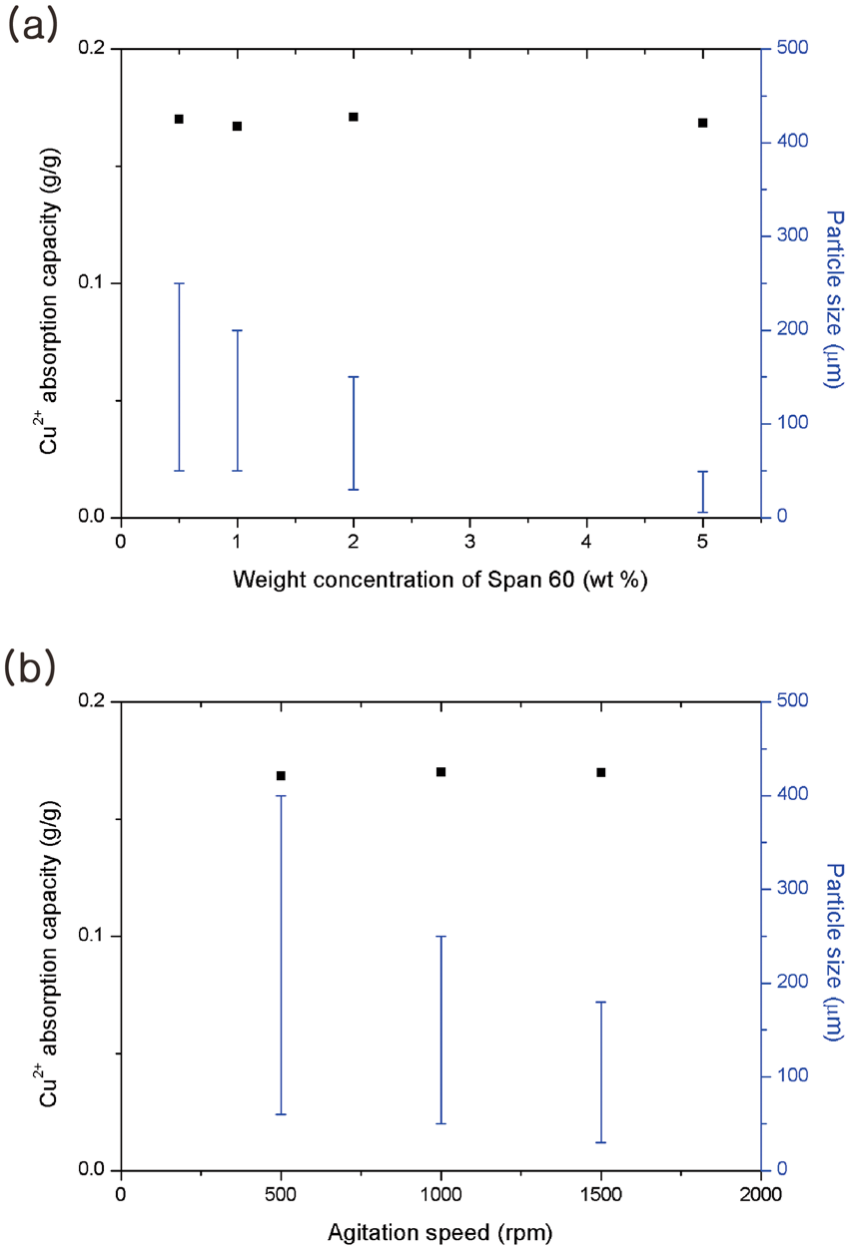
Effect of (**a**) the amount of stabilizer and (**b**) the agitation speed on the Cu^2+^ absorption capacity and particle size.

**Figure 5:**
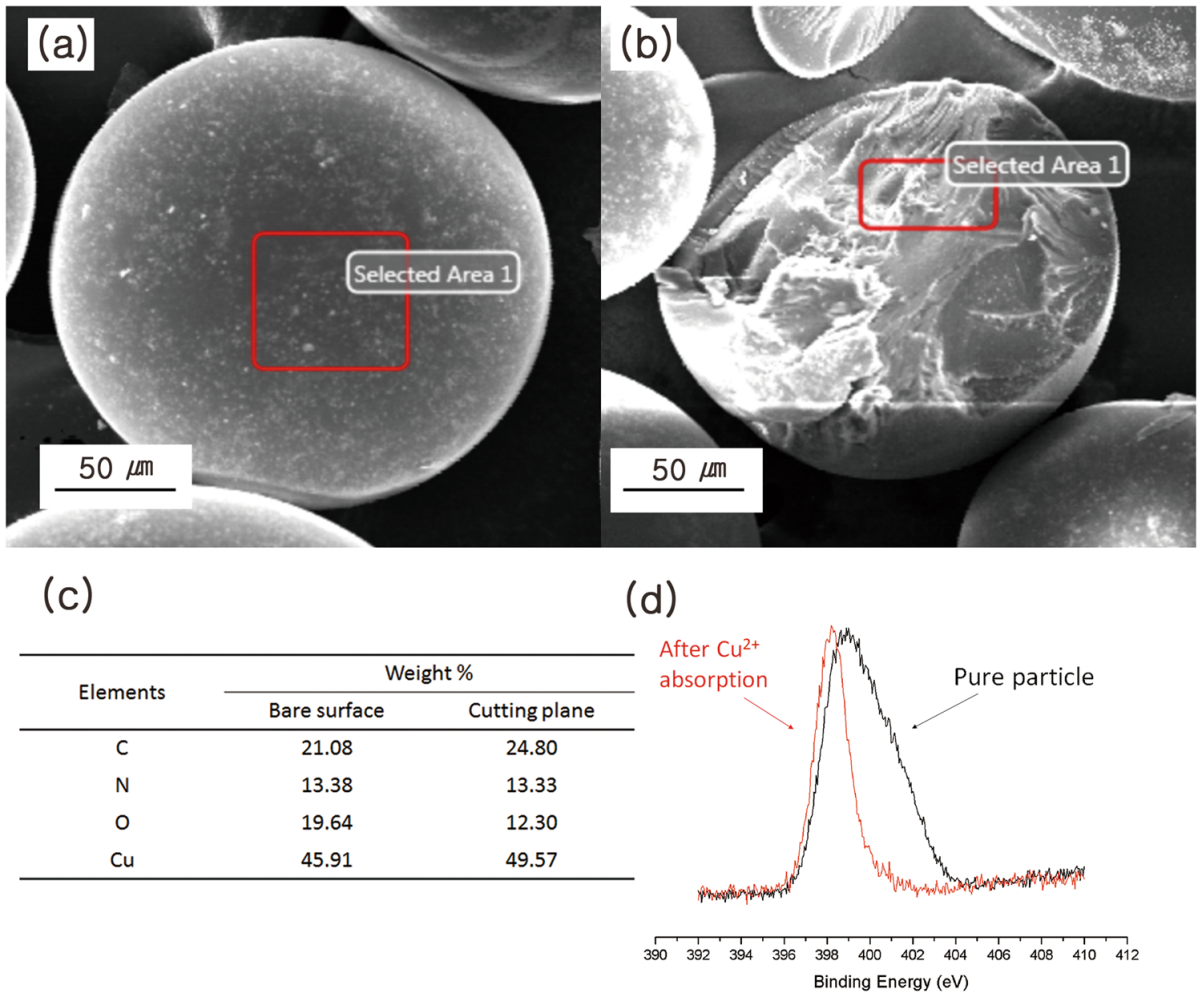
EDX and XPS analyses of HPAMAM particles of **P-M**_**12/8**_ **S**_**0.5**_ **R**_**1k**_ after Cu^2+^ absorption: (**a**) collected area on the bare surface, (**b**) collected area on the cutting plane, (**c**) element analysis data, and (**d**) XPS spectra of the N_1s_ region of **P-M**_**12/8**_ **S**_**0.5**_ **R**_**1k**_ before (black line) and after (red line) Cu^2+^ absorption.

Interestingly, the particle sizes that varied with the amount of stabilizer and the speed of agitation did not affect the swelling ratio or the Cu^2+^ absorption capacity of the particles (Fig. 4 and Fig. S9, ESI), presumably because the binding of metal ions occurred not only on the surface but also in the inside of the particles. An energy dispersive X-ray spectroscopy (EDX) analysis of the cutting plane of the particles clearly showed the presence of Cu atoms inside (Fig. 5). Greater absorption of metal ions by the particles was realized through the suction of metal ions into the inside of particles fully consisting of HPAMAM.

X-ray photoelectron spectroscopy (XPS) results showed decreased binding energy of N_1s_ upon Cu^2+^ absorption by the particles, as shown in Fig. 5(d). As described in the literature, chelation between the electron-rich amine and amide groups with electron-deficient metal atoms is the key principle of absorption^46, 47^.

**Figure 6:**
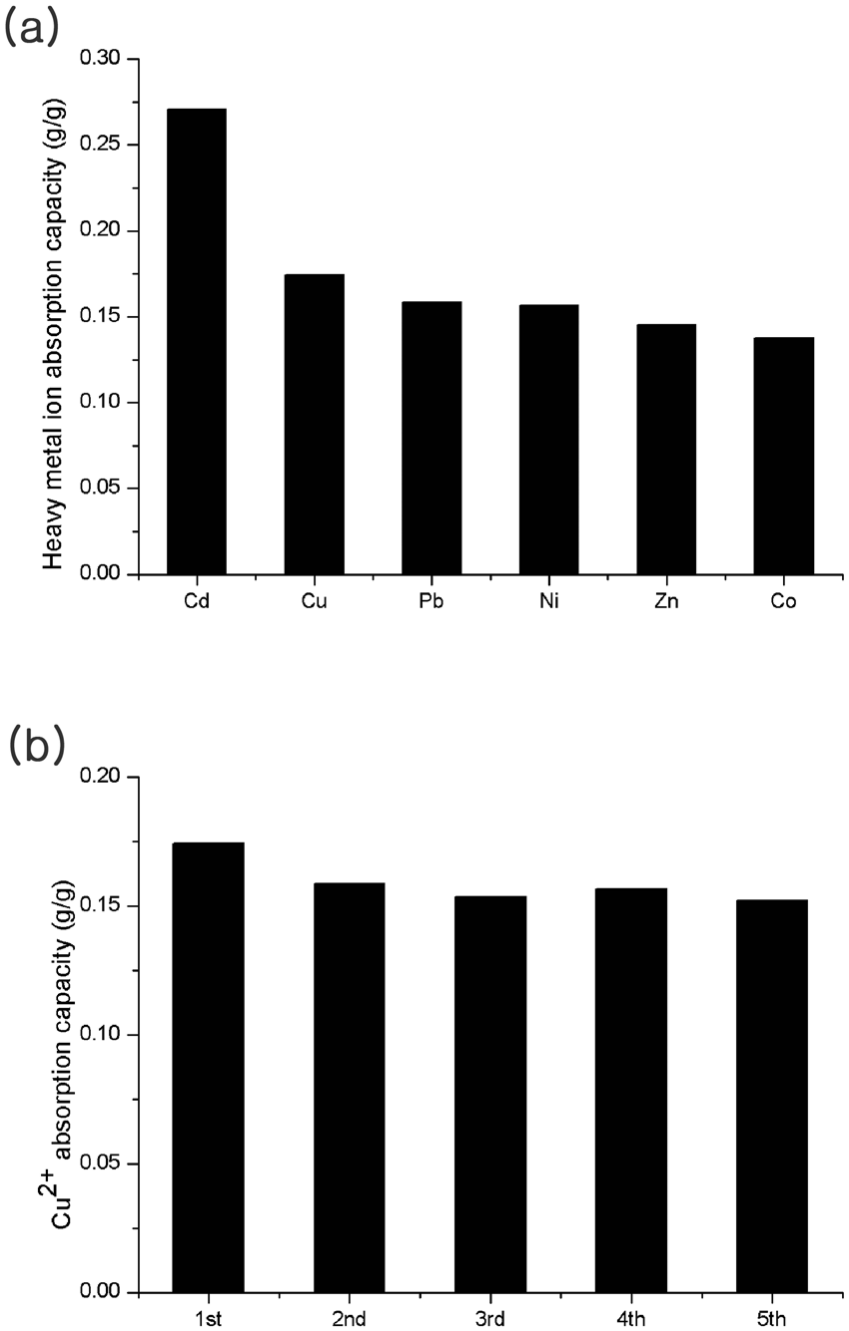
(**a**) Absorption capacities of various heavy metal ions and (**b**) a recycle test of **P-M**_**8/8**_ **S**_**0.5**_ **R**_**1k**_.

The absorption capacity of other heavy metal cations, including Cd^2+^, Cu^2+^, Pb^2+^, Ni^2+^, Zn^2+^, and Co^2+^, were studied with **P-M**_**8/8**_ **S**_**0.5**_ **R**_**1k**_. The HPAMAM particles showed excellent metal absorption capacities (g/g) of more than 0.13 with all of the heavy metals tested in this study. An especially outstanding result was noted with Cd^2+^ (0.27 g/g, Fig. 6). HPAMAM particles have excellent Cu^2+^ and Cd^2+^ absorption capacities compared to those of other different types of absorbents reported recently (Table 1 and Table S2, ESI). The PAMAM dendrimer has better Cu^2+^ absorption capacity, but these dendrimers are difficult to apply owing to the costly synthesis and the additional separation process.

**Table 1:** Cu^2+^ and Cd^2+^ Absorption capacities of different absorbents.

Metal ions	Absorbent	Absorption capacities (mg/g)	Reference
Cu^2+^	Mesoporous carbon	24	47
	Polyaniline graft chitosan beads	100	48
	Dowex M4195	54	49
	PAMAM dendrimer (M.W. 14,000)	329	25
	HPAMAM particle	170	This work
Cd^2+^	Linde type A zeolite	147	26
	Amberjet 1200 H	130	50
	PAMAM dendrimer (M.W. 14,000)	169	26
	HPAMAM particle	270	This work

The reusability of particles for heavy metal absorption was investigated with **P-M**_**8/8**_ **S**_**0.5**_ **R**_**1k**_ (Fig. 6(b)). The desorption of Cu^2+^ was easily achieved through a treatment with a 0.1 N HCl solution. At a low pH, the chelation between metal ions and nitrogen atoms is weakened because competitive H^+^ ions occupy the amine groups, whereas the protonated amine groups are deprotonated with an increase in the pH value. During the first recycle step, the Cu^2+^ absorption capacity declined to around 0.02 degrees, presumably due to the undetached copper ions which arose during the desorption process. Nevertheless, recycled HPAMAM particles showed excellent Cu^2+^ absorption capacities which exceeded 0.15 with up to five times of recycling.

In order to investigate the possibility of mass production, a scale up test was attempted. When the experimental scale was increased to 10 L of the organic phase, spherical polymer particles 50~250 μm in size were obtained steadily with the expected yield. All of the PAMAM particles showed a consistent swelling ratio and Cu^2+^ an absorption capacity which exceeded 0.15 (Table S3 and Fig. S10, ESI).

**Figure 7:**
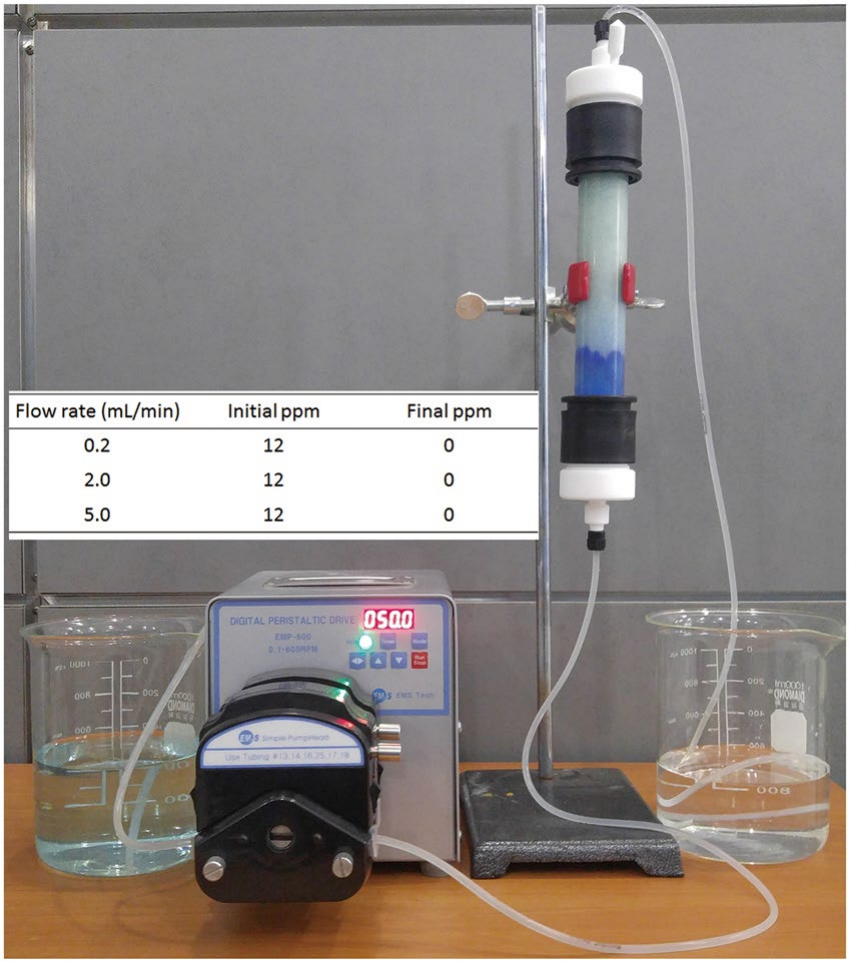
Demonstration of a metal ion absorption test using a column packed with **P-M**_**12/8**_ **S**_**0.5**_ **R**_**0.3k**_ **-500** particles (inset: in and out concentrations of Cu^2+^ in water with various flow rates).

Copper ion removal by the micro-hydrogel particles packed within a glass column (a **P-M**_**12/8**_ **S**_**0.5**_ **R**_**0.3k**_ **−500** particle-packed column (Fig. 7)) was examined with a continuous flow of a Cu^2+^ solution. With the flow of the Cu^2+^ solution, PAMAM particles absorbed Cu^2+^ ions in the aqueous solution, and their color became blue starting from the lower part of the column. Even under a high flow rate (5.0 mL/min), the Cu^2+^ ions in the aqueous solution were completely removed up to the limit of detection.

In summary, micro-hydrogel particles consisting wholly of HPAMAM were readily synthesized by an eco-friendly polyaddition reaction of A_2_ and B_4_ types of monomers via inverse suspension polymerization. The particles were found to be highly efficient during the removal of heavy metal ions, in this case cadmium, copper, lead, nickel, zinc, and cobalt, from water. Cu^2+^ absorption capacity was found to be as high as 0.17 g/g, which is three times that of other polystyrene-based chelating polymer adsorbents. This superb absorption capacity stems from the good hydrophilic properties and high density levels of the amine and amide functional groups without any supporting materials. In addition, easy recycling through control of the pH, a successful scale-up operation, as well as the complete removal of metal ions from water through a column packed with the HPAMAM particles demonstrate that the crosslinked HPAMAM particles, obtained by an eco-friendly process, can serve as a cost-effective and efficient absorbent for water purification applications.

## Methods

### Polymerization of HPAMAM

EDA (0.5259 g; 8.75 mmol), MBA (1.3491 g; 8.75 mmol), and water (3.75 mL; 50 wt/v% of the monomers) were placed in a round bottomed-flask (RBF) and stirred for 6 h at 60 °C under nitrogen atmosphere. The reaction mixture was cooled to room temperature, and precipitated into acetone. The supernatant was drained out of the vial and the remaining sticky polymer was dried under vacuum for 24 h at 60 °C. Yield = 90%.

### Polymerization Kinetics of HPAMAM

The polymerization kinetics of HPAMAM was studied by monitoring the polymerization with^1^H NMR. ^1^H NMR spectroscopy a were recorded on a Bruker Fourier Transform Avance 400 (400 MHz) spectrometer. A typical procedure is as follows. EDA(0.8415 g; 14.00 mmol), MBA (2.1585 g; 14.00 mmol) and deuterium oxide (10 mL; 30 wt% of the monomers) were placed in a RBF and stirred for 6 h at 60 °C^1^.H NMR spectra were recorded at 0, 5, 10, 20, 30, 40, 50, 60 min.

### Synthesis of HPAMAM Particles by Inverse Suspension Polymerization

**P-M**_**8/8**_ **S**_**0.5**_ **R**_**1k**_ The inverse suspension polymerization reactions were carried out as follows. The suspension stabilizer Span^®^ 60 (0.0094 g; 0.5 wt% of the monomers) was dissolved in toluene (15 mL; 4 times of water) and degassed for 30 min under vigorous agitation. EDA (0.5259 g; 8.75 mmol), MBA (1.3491 g; 8.75 mmol), and water (3.75 mL; 50 wt/v% of the monomers) were placed and stirred in a RBF until complete dissolution of the solid. The aqueous solution was introduced into the toluene solution for inverse suspension polymerization at 45 °C under nitrogen with the stirring speed of 1,000 rpm. After 6 hr of polymerization, the polymerization mixture was poured into methanol, filtered and washed several times alternatively with methanol and acetone, and then dried under vacuum for 24 hr at 60 °C.

**P-M**_**9/8**_ **S**_**0.5**_ **R**_**1k**_ **to P-M**_**16/8**_ **S**_**0.5**_ **R**_**1k**_ P-M_9/8_S_0.5_R_1k_ – P-M_16/8_S_0.5_R_1k_ were prepared according to the method for P-M_8/8_S_0.5_R_1k_ except the feed composition of MBA and EDA. The molar ratios of the monomers (MBA/EDA) were 1.125, 1.25, 1.375, 1.5, 1.625, 1.75, 1.875, and 2.0, to produce P-M_9/8_S_0.5_R_1k_, P-M_10/8_S_0.5_R_1k_, P-M_11/8_S_0.5_R_1k_, P-M_12/8_S_0.5_R_1k_, P-M_13/8_S_0.5_R_1k_, P-M_14/8_S_0.5_R_1k_, P-M_15/8_S_0.5_R_1k_, and P-M_16/8_S_0.5_R_1k_ respectively.

### Scale Up Test

**P-M**_**12/8**_ **S**_**0.5**_ **R**_**1k**_ **-30, 100, 150, 300, P-M**_**12/8**_ **S**_**0.5**_ **R**_**0.3k**_-**500, 750, 2000, 10000** Toluene – 30–10000 mL scale were prepared according to the method for P-M_8/8_S_0.5_R_1k_, except the amount of reactant. The amount of each reactant was a fixed ratio. P-M_12/8_S_0.5_R_1k_-30, 100, 150, 300 were stirred by stirring bar at 1,000 rpm. P-M_12/8_S_0.5_R_0.3k_-500, 750, 2000, 10000 were stirred by mechanical stirrer at 300 rpm.

## Electronic supplementary material

Supplementary Information 
