# Supplementary material for: Micro-hydrogel Particles Consisting of Hyperbranched Polyamidoamine for the Removal of Heavy Metal Ions from Water
Source: Sci Rep. 2017 Aug 30;7:10012. doi: 10.1038/s41598-017-10066-x (PMC5577261; doi:10.1038/s41598-017-10066-x)
Supplement: Supplementary file 1 — Supplementary Information [file 41598_2017_10066_MOESM1_ESM.pdf]

Electronic Supplementary Information (ESI)

Micro-hydrogel Particles Consisting of Hyperbranched Polyamidoamine for the Removal of Heavy Metal Ions from Water

Sanghwa Lee,[a] Youngsik Eom,[a] Jeyoung Park,[b,c] Jinhee Lee,[a] Sang Youl Kim*[a]

[a] Department of Chemistry, Korea Advanced Institute of Science and Technology (KAIST), Daejeon, 34141, Republic of Korea.

[b] Research Center for Bio-based Chemistry, Korea Research Institute of Chemical Technology (KRICT), Ulsan, 44429, Republic of Korea

[c] Advanced Materials and Chemical Engineering, University of Science & Technology (UST), Daejeon, 34113, Republic of Korea

* To whom correspondence should be addressed. Professor S. Y. Kim, Tel: (+) 82-42-350-2834, Fax: (+) 82-42-350-8177, E-mail: kimsy@kaist.ac.kr

General Information

All chemicals were purchased from Sigma-Aldrich. The materials used in the inverse suspension polymerization experiments were ethylenediamine (EDA), N,N'-methylenebisacrylamide (MBA), sorbitan monostearate (Span® 60), and toluene . All reactants were used as received without further purification. EDA and MBA are monomers for Michael addition reaction, and Span 60 is a suspension stabilizer. Also, $CdCl_2$, $CuCl_2$, $PbCl_2$, $NiCl_2$, $ZnCl_2$, $CoCl_2$ were used for the preparation of Cd(II), Cu(II), Pb(II), Ni(II), Zn(II), Co(II) stock solutions.

^{1}H NMR spectra were recorded on a Bruker Fourier Transform Avance 400 spectrometer for the polymers dissolved in deuterium oxide. FT-IR spectra were obtained with a Bruker EQUINOX-55 spectrometer. Thermal stability studies of the particles were carried out using a TA 2200 thermal analyzer system with a scan rate of 10 ^{o}C min^{-1} under a flow nitrogen gas. The morphology of the polymer particles was examined by optical microscopy (OM) and scanning electron microscopy (SEM). Copper ion binding capacities were measured by inductively coupled plasma optical emission spectrometer (Agilent ICP-OES 720).

Measurement of Swelling Ratio

We measured the swelling ratio (Q) by following procedure. The dry HPAMAM particles (W_{dry}) were placed in distilled water and kept there for at least 2 days to reach swelling equilibrium at room temperature. Equilibrated swollen HPAMAM particles were then removed from water and tapped with filter paper to dry the particle surface. And then the particles were collected and weighted (W_s). Swelling ratio (Q) was calculated from the following equation (1).

$$\text{Swelling ratio (Q)} = [(W_s - W_{dry})/W_{dry}] \quad (1)$$

where W_{dry} and W_s are the weights of the dry sample and the swollen particles, respectively.

Measurement of Copper Ion Adsorption Capacity

Binding experiments were carried out in batches as follow. Stock copper solution was prepared as 10,000 ppm in distilled deionized water. The dry HPAMAM particles were placed in the stock heavy metal solution and kept there for at least 1 days to reach equilibrium at room temperature. The HPAMAM particles were separated from the metal solutions by filtration with cellulose nitrate membrane filters with a pore size of 0.45 μm. The concentration of metals in the filtrate was determined by using inductively coupled plasma optical emission spectrometer (Agilent ICP-OES 720). Copper ion binding capacity (A) was calculated from the following equation (2).

$$\text{Copper ion binding capacity (A)} = [(C_0 - C_e)V/m] \quad (2)$$

where m (g) is the weights of the dry sample, V (L) is the volume of the stock copper solution, C_0 and C_e (g/L) are the initial and equilibrium copper ion concentrations, respectively.

Measurement of Gel Fraction

The dry HPAMAM particles (W_{before}) were placed in distilled water and stirred for at least 2 days to dissolve unreacted monomers and oligomers at room temperature. HPAMAM particles were then removed from water and tapped with filter paper and then dried under vacuum for 24hr at 60 ^{o}C. Then the weight of dry HPAMAM particles were measured (W_{after}). Gel fraction was calculated from the following equation (3).

$$\text{Gel fraction} = (W_{after}/W_{before})*100 \quad (3)$$

Metal Ion Adsorption Test Using Column Method

Glass column (Spectra/Chrom^{TM} LC Column, diameter: 2.5 cm, volume: 4.91ml/cm) was prepared for our fixed-bed column adsorption test. 25g of P-$M_{12/8}S_{0.5}R_{0.3k}$-500 particles were

placed in glass column. In order to pack the PAMAM particles in glass column, water was injected to percolate through the column at the flow rate of 2.0 mL/min. Then, Cu^{2+} aqueous solution was allowed to flow at each flow rate.

EDX analysis

Frozen HPAMAM particles with liquid nitrogen were broken by spatula, then cut HPAMAM particles were prepared for element analysis of inner part of HPAMAM particles. Element composition was determined by Hitachi SU8230.

XPS analysis

HPAMAM particles, Cu^{2+} adsorbed HPAMAM particles, and Cu^{2+} solution were prepared on silicon wafer. XPS spectra were recorded by Thermo VG Scientific Sigma Probe.

Table S1. Properties of PAMAM particles

	[MBA]/[EDA] [a]	Span 60 (wt%) [b]	Agitation speed (rpm) [c]	Size (μm) [d]	Gel fraction (%) [e]	Swelling ratio (g/g) [f]	Cu^{2+} adsorption capacity (g/g) [g]
P-$M_{8/8}S_{0.5}R_{1k}$	1.000	0.5	1000	50~250	88.020	11.017	0.199
P-$M_{9/8}S_{0.5}R_{1k}$	1.125			50~250	96.298	5.198	0.188
P-$M_{10/8}S_{0.5}R_{1k}$	1.250			50~250	97.514	3.637	0.176
P-$M_{11/8}S_{0.5}R_{1k}$	1.375			50~250	97.251	3.184	0.163
P-$M_{12/8}S_{0.5}R_{1k}$	1.500			50~250	97.908	2.870	0.156

P-$M_{13/8}S_{0.5}R_{1k}$	1.625			50~250	97.968	2.374	0.138
P-$M_{14/8}S_{0.5}R_{1k}$	1.750			50~250	97.741	2.143	0.125
P-$M_{15/8}S_{0.5}R_{1k}$	1.875			50~250	98.852	2.015	0.110
P-$M_{16/8}S_{0.5}R_{1k}$	2.000			50~250	99.102	2.027	0.109
P-$M_{12/8}S_{1.0}R_{1k}$		1.0		50~200	-	2.889	0.167
P-$M_{12/8}S_{2.0}R_{1k}$		2.0		30~150	-	3.005	0.171
P-$M_{12/8}S_{5.0}R_{1k}$	1.500	5.0		5~50	-	2.844	0.168
P-$M_{12/8}S_{0.5}R_{0.5k}$		0.5	500	60~400	-	2.934	0.169
P-$M_{12/8}S_{0.5}R_{1.5k}$			1500	30~180	-	3.002	0.170

[a] Initial molar feed ratio of MBA to EDA([MBA]/[EDA]). [b] Weight concentration of Span 60 (Wt%). [c] Agitation speed (rpm). [d] Size range of micro-hydrogel particles (μm). [e] Gel fraction (%). [f] Swelling ratio (g/g). [g] Cu^{2+} adsorption capacity (g/g).

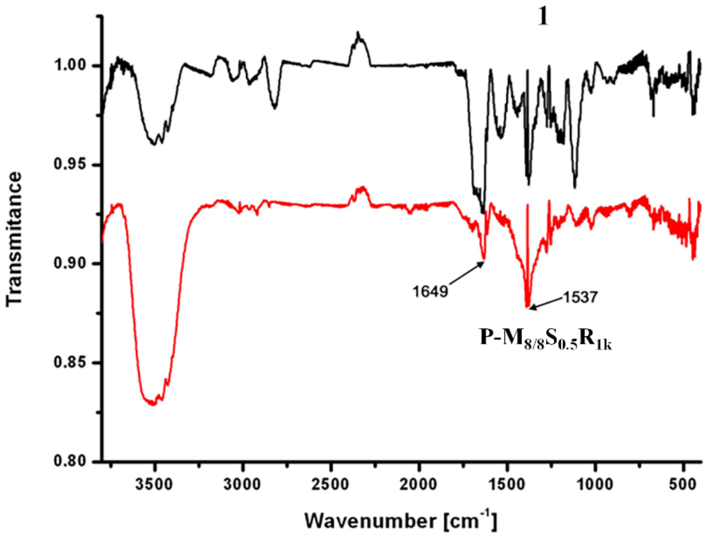

Fig. S1 FT-IR spectra for **1** and P-$M_{8/8}S_{0.5}R_{1k}$

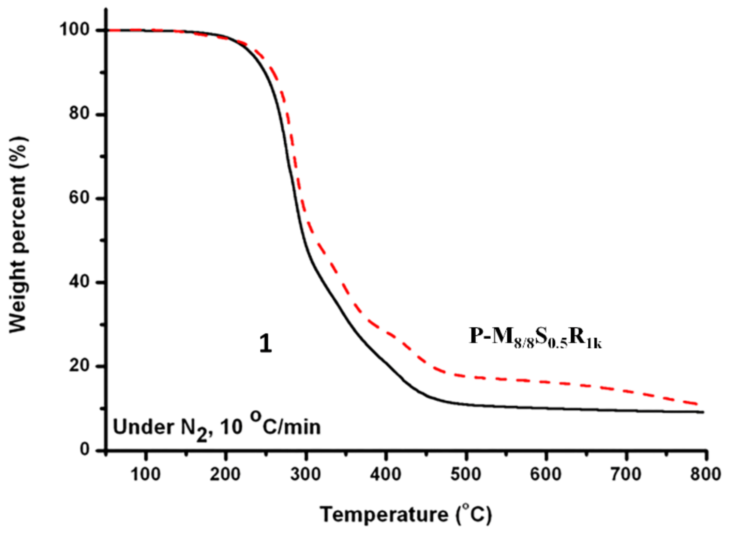

Fig. S2 Thermogravimetic analysis

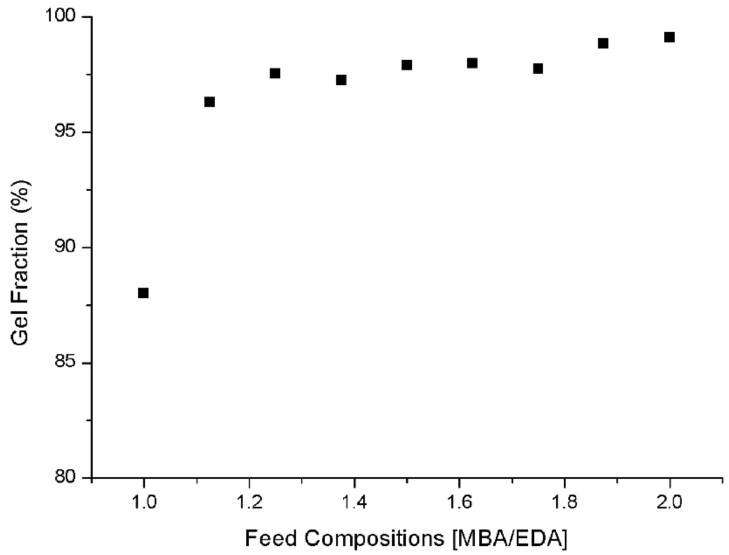

Fig. S3 Gel fraction of HPAMAM particles with feed compositions

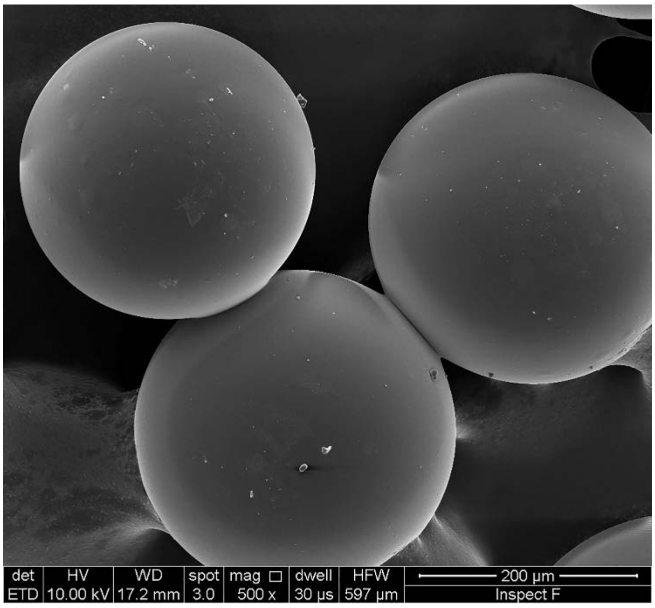

Fig. S4 SEM image of P-$M_{8/8}S_{0.5}R_{1k}$

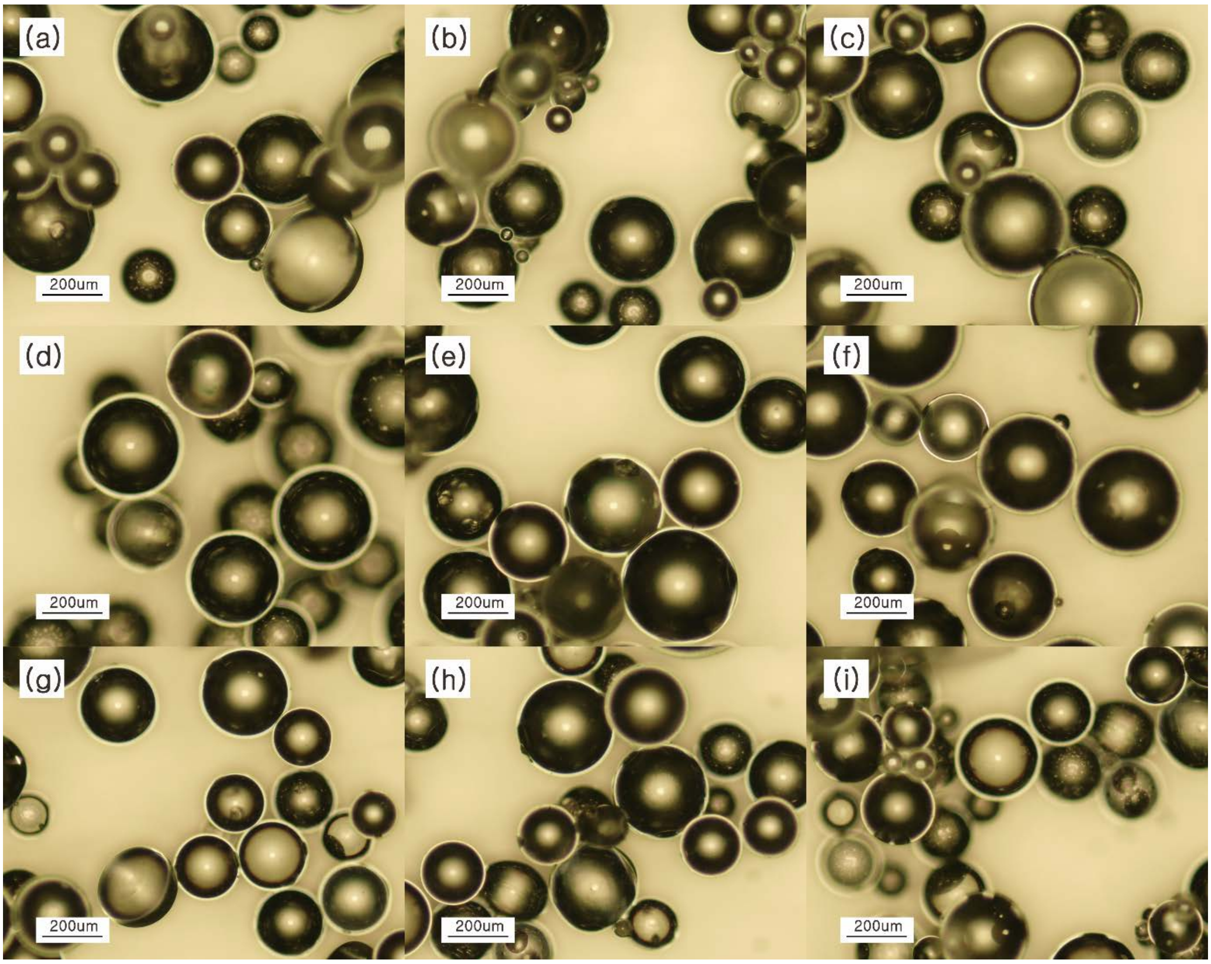

Fig. S5 OM images of (a) P-$M_{8/8}S_{0.5}R_{1k}$; (b) P-$M_{9/8}S_{0.5}R_{1k}$; (c) P-$M_{10/8}S_{0.5}R_{1k}$; (d) P-$M_{11/8}S_{0.5}R_{1k}$; (e) P-$M_{12/8}S_{0.5}R_{1k}$; (f) P-$M_{13/8}S_{0.5}R_{1k}$; (g) P-$M_{14/8}S_{0.5}R_{1k}$; (h) P-$M_{15/8}S_{0.5}R_{1k}$; (i) P-$M_{16/8}S_{0.5}R_{1k}$

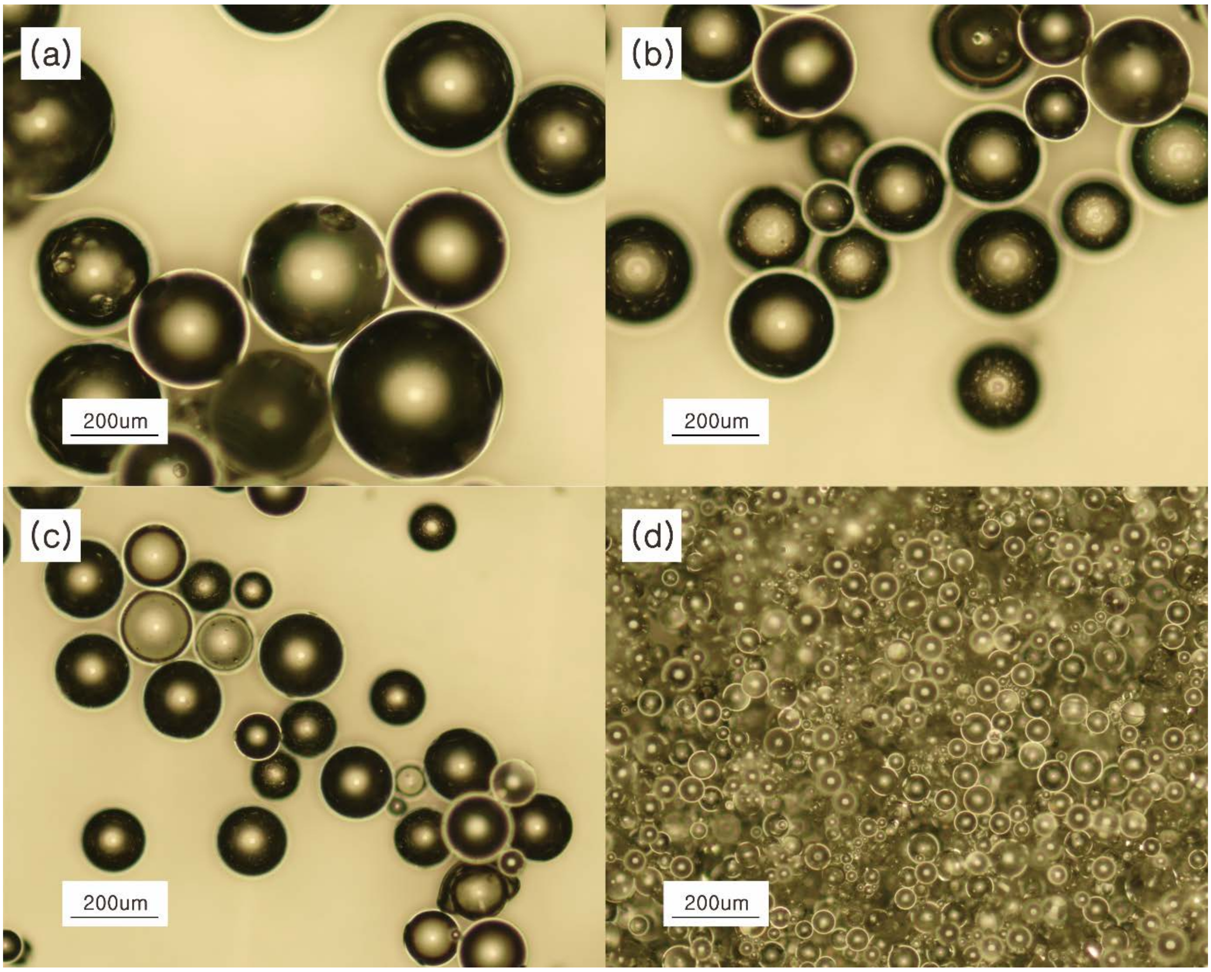

Fig. S6 OM images of (a) P-$M_{12/8}S_{0.5}R_{1k}$; (b) P-$M_{12/8}S_{1.0}R_{1k}$; (c) P-$M_{12/8}S_{2.0}R_{1k}$; (d) P-$M_{12/8}S_{5.0}R_{1k}$;

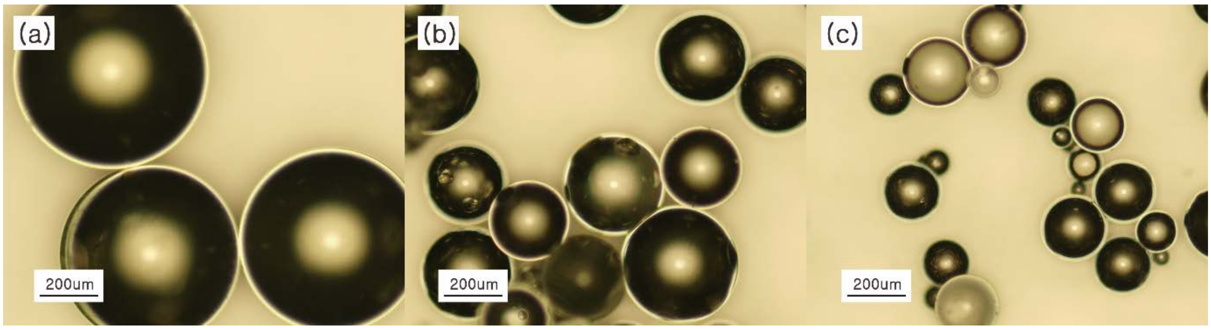

Fig. S7 OM image of (a) P-$M_{12/8}S_{0.5}R_{0.5k}$; (b) P-$M_{12/8}S_{0.5}R_{1k}$; (c) P-$M_{12/8}S_{0.5}R_{1.5k}$;

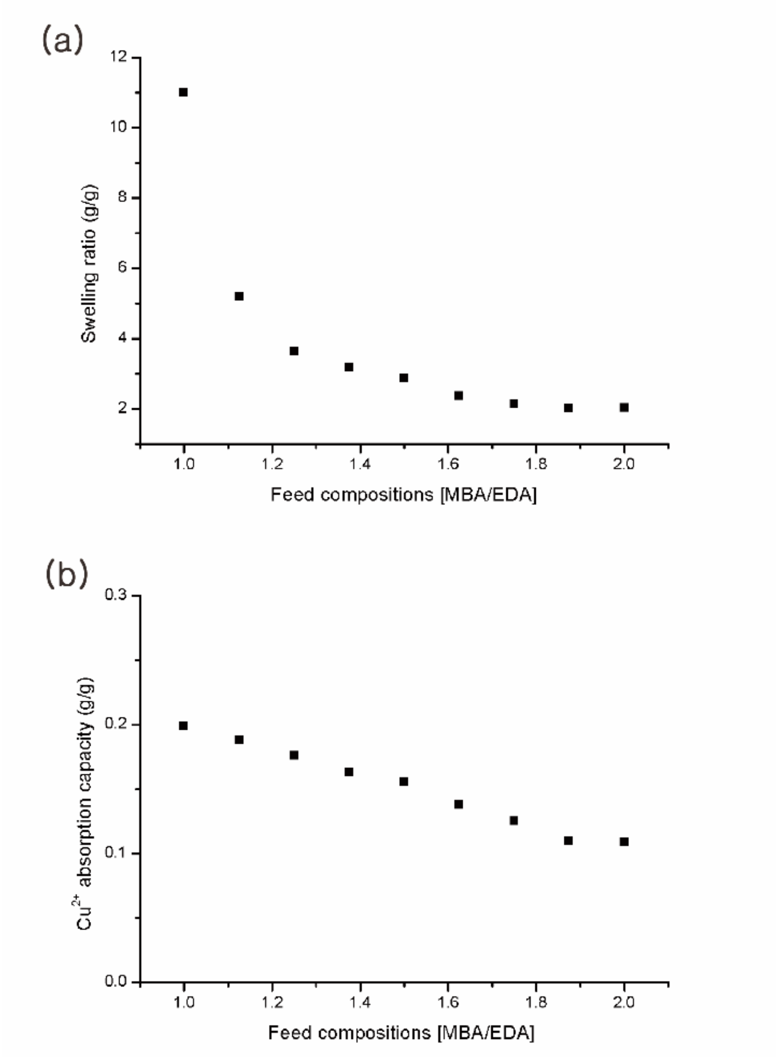

Fig. S8 Effect of feed compositions of monomers on (a) swelling ratio and (b) Cu^{2+} absorption

capacity

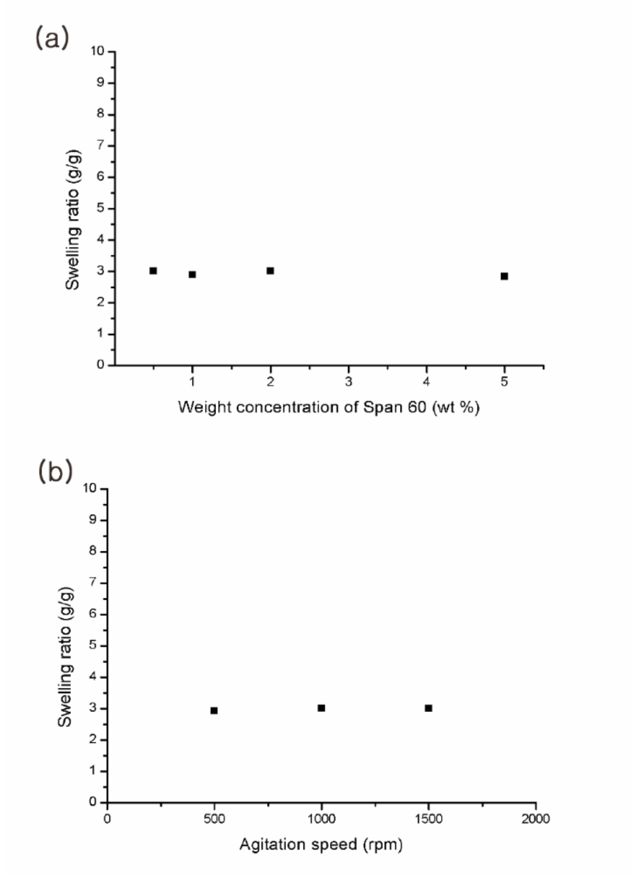

Fig. S9 Effect of amount of stabilizer (a) and agitation speed (b) on a swelling ratio

Table S2 Cu^{2+} Absorption capacities of different adsorbents

Absorbent	Absorption capacities (mg/g)	Ref
Anatase nanoabsorbent	23.74	1
Mesoporous carbon	56.62	2
Polystyrene-supported chitosan	99.8	3
Polyaniline graft chitosan beads	100	4
Polyacrylonitrile fiber functionalized iminodiacetic acid	119.39	5
Poly(vinylbenzyl chloro-*co*-styrene-*co*-divinyl benzene) bead	61.2	6
N(2-sulfoethyl) chitosan	99.8	7
Carboxylic acid functionalized poly(glycidyl methacrylate)	37.5	8
Deacetylated konjac glucomannan confugated soy protein isolate	62.5	9
DowexTM M4195	54	
Purolite TM S-930Plus	48	
Diaion TM Cr11	48	

Table S3. Properties of PAMAM and PAMAM particles

	Volume of organic phase (mL) [a]	Span 60 (wt%) [b]	Agitation speed (rpm) [c]	Size (μm) [d]	Swelling ratio (g/g) [e]	Cu^{2+} absorption capacity (g/g) [f]
P-$M_{12/8}S_{0.5}R_{1k}$-15	15	0.5	1000	50~250	2.870	0.156
P-$M_{12/8}S_{0.5}R_{1k}$-30	30				2.905	0.160
P-$M_{12/8}S_{0.5}R_{1k}$-100	100				3.103	0.169
P-$M_{12/8}S_{0.5}R_{1k}$-150	150				3.052	0.175
P-$M_{12/8}S_{0.5}R_{0.3k}$-300	300				2.958	0.169
P-$M_{12/8}S_{0.5}R_{0.3k}$-500	500		300		3.153	0.181
P-$M_{12/8}S_{0.5}R_{0.3k}$-750	750				3.033	0.172
P-$M_{12/8}S_{0.5}R_{0.3k}$-2000	2000				3.019	0.178
P-$M_{12/8}S_{0.5}R_{1k}$-10000	10000				2.974	0.171

[a] Volume of organic solvent (mL). [b] Weight concentration of Span 60 (Wt%). [c] Agitation speed (15~300–stirring bar, 500~10000-mechanical stirrer); (rpm). [d] Size range of micro-hydrogel particles (μm). [e] Swelling ratio (g/g). [f] Cu^{2+} adsorption capacity (g/g).

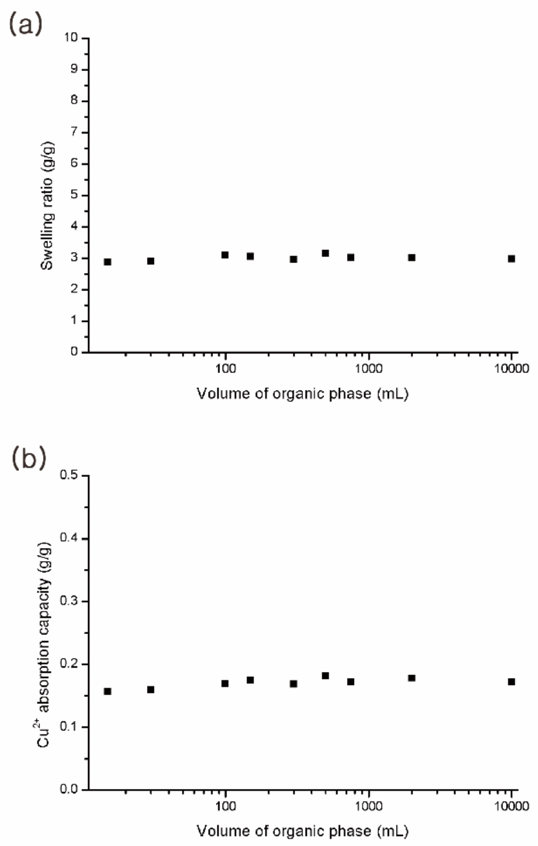

Fig. S10 Effect of the reaction scale on swelling ratio (a) and Cu^{2+} absorption capacity (b)

Reference

1. Özlem Kocabaş-Atakli, Z. & Yürüm, Y. Synthesis and characterization of anatase nanoadsorbent and application in removal of lead, copper and arsenic from water. *Chem. Eng. J.* **225,** 625–635 (2013).

2. Huang, C.-C. & He, J.-C. Electrosorptive removal of copper ions from wastewater by using ordered mesoporous carbon electrodes. *Chem. Eng. J.* **221,** 469–475 (2013).

3. Jiang, W. *et al.* Spherical polystyrene-supported chitosan thin film of fast kinetics and high capacity for copper removal. *J. Hazard. Mater.* **276,** 295–301 (2014).

4. Igberase, E., Osifo, P. & Ofomaja, A. The adsorption of copper (II) ions by polyaniline graft chitosan beads from aqueous solution: Equilibrium, kinetic and desorption studies. *J. Environ. Chem. Eng.* **2,** 362–369 (2014).

5. Deng, S., Zhang, G., Wang, X., Zheng, T. & Wang, P. Preparation and performance of polyacrylonitrile fiber functionalized with iminodiacetic acid under microwave irradiation for adsorption of Cu(II) and Hg(II). *Chem. Eng. J.* **276,** 349–357 (2015).

6. Lapwanit, S., Trakulsujaritchok, T. & Nongkhai, P. N. Chelating magnetic copolymer composite modified by click reaction for removal of heavy metal ions from aqueous solution. *Chem. Eng. J.* **289,** 286–295 (2016).

7. Petrova, Y. S., Pestov, A. V., Usoltseva, M. K. & Neudachina, L. K. Selective adsorption of silver(I) ions over copper(II) ions on a sulfoethyl derivative of chitosan. *J. Hazard. Mater.* **299,** 696–701 (2015).

8. Han, J., Du, Z., Zou, W., Li, H. & Zhang, C. In-situ improved phenol adsorption at ions-enrichment interface of porous adsorbent for simultaneous removal of copper ions

and phenol. *Chem. Eng. J.* **262,** 571–578 (2015).

9. Liu, F. *et al.* Removal of copper(II) using deacetylated konjac glucomannan conjugated soy protein isolate. *Int. J. Biol. Macromol.* **86,** 338–344 (2016).
